# Salivary Cortisol Determination on Smartphone-Based Differential Pulse Voltammetry System

**DOI:** 10.3390/s20051422

**Published:** 2020-03-05

**Authors:** Jingjing Liu, Ning Xu, Hong Men, Shuang Li, Yanli Lu, Sze Shin Low, Xin Li, Lihang Zhu, Chen Cheng, Gang Xu, Qingjun Liu

**Affiliations:** 1 Biosensor National Special Laboratory, Key Laboratory for Biomedical Engineering of Education Ministry, Department of Biomedical Engineering, Zhejiang University, Hangzhou 310027, China; jingjing_liu@neepu.edu.cn (J.L.); lishuangv@tju.edu.cn (S.L.); yanlilu@zju.edu.cn (Y.L.); szeshin.low@zju.edu.cn (S.S.L.); 21815060@zju.edu.cn (X.L.); zhulihang@zju.edu.cn (L.Z.); chen_cheng@zju.edu.cn (C.C.); gxu@zju.edu.cn (G.X.); 2 College of Automation Engineering, Northeast Electric Power University, Jilin 132012, China; 2201600442@neepu.edu.cn (N.X.); menhong@neepu.edu.cn (H.M.); 3 Department of Computer Science and Bioimaging Research Center, University of Georgia, Athens, GA 30602, USA

**Keywords:** salivary cortisol, immunosensor, molybdenum disulfide, gold nanoparticles, differential pulse voltammetry, smartphone

## Abstract

Cortisol is commonly used as a significant biomarker of psychological or physical stress. With the accelerated pace of life, non-invasive cortisol detection at the point of care (POC) is in high demand for personal health monitoring. In this paper, an ultrasensitive immunosensor using gold nanoparticles/molybdenum disulfide/gold nanoparticles (AuNPs/MoS_2_/AuNPs) as transducer was explored for non-invasive salivary cortisol monitoring at POC with the miniaturized differential pulse voltammetry (DPV) system based on a smartphone. Covalent binding of cortisol antibody (CORT-Ab) onto the AuNPs/MoS_2_/AuNPs transducer was achieved through the self-assembled monolayer of specially designed polyethylene glycol (PEG, SH-PEG-COOH). Non-specific binding was avoided by passivating the surface with ethanolamine. The miniaturized portable DPV system was utilized for human salivary cortisol detection. A series current response of different cortisol concentrations decreased and exhibited a linear range of 0.5–200 nM, the detection limit of 0.11 nM, and high sensitivity of 30 μA M^−1^ with a regression coefficient of 0.9947. Cortisol was also distinguished successfully from the other substances in saliva. The recovery ratio of spiked human salivary cortisol and the variation of salivary cortisol level during one day indicated the practicability of the immunosensor based on the portable system. The results demonstrated the excellent performance of the smartphone-based immunosensor system and its great potential application for non-invasive human salivary cortisol detection at POC.

## 1. Introduction

With the development of society, intense competition and increasing levels of stress, many chronic diseases such as depression, post-traumatic stress disorder (PTSD), heart attack, and brain pain have become a serious concern, challenging the life of developed countries [1,2,3,4]. Cortisol is a glucocorticoid class hormone that is stimulated by the adrenocorticotrophic hormone and secreted from the adrenal glands located above the kidneys. It plays a major part in regulating a variety of physiological processes such as blood pressure, glucose levels, and carbohydrate metabolism, as well as human cognitive behavior like memory, sleep condition and mood, making it a relevant biomarker for the detection of numerous psychological stress-related diseases and fatigue monitoring [5,6,7]. Abnormal levels of cortisol can lead to inflammation, a fragile immune system, and increased fat and amino acid levels in blood. Levels of cortisol above or below the normal values have been related to the development of Cushing’s disease and Addison’s disease respectively [8,9,10]. In addition, there is a circadian rhythm for cortisol secretion during a day, with cortisol level the highest in the morning and then gradually lower [11,12]. Therefore, regular monitoring of cortisol level is deemed essential for timely diagnostics and treatment. Hence, it is meaningful to establish a miniaturized and portable detection system for non-invasive cortisol investigation at POC. 

Nowadays, the smartphone has become an indispensable mobile electronic device in people’s daily life worldwide due to their portability, internet connection, powerful data-processing ability and easy operation. The merits of smartphones brought new possibilities for biomedical and biosensor research. Recently, many studies on smartphone-controlled platforms at the point of care (POC) have been published and several electrochemical methods have been combined with smartphone for quantitative electrochemical detections, such as chronoamperometry (CA), cyclic voltammetry (CV), differential pulse voltammetry (DPV) [13,14] and electrochemical impedance spectroscopy (EIS) [14,15,16,17]. Among them, DPV is capable of detection of low-level concentrations of analytes. Some portable devices have been explored for a low-level detection range of cortisol [18,19,20]. However, few studies have exploited smartphone-controlled devices for non-invasive and POC salivary cortisol detection. This requires higher requirements for the performance of a cortisol sensor. Thus, it is crucial to establish a suitable sensing platform with unique properties to cooperate with the smartphone-controlled DPV system to detect salivary cortisol. 

Meanwhile, current clinical estimation of cortisol mainly includes fluorescent enzyme-linked immunosorbent assays (ELISA) [21], radioimmunoassays (RIA) [22], traditional liquid chromatography tandem mass spectrometry (LC-MS) [23], fluorometric assays [24], and so on. They perceive the merits of high sensitivity and specificity to the analytes, but they are laborious, time-consuming, expensive, require large amounts of sample, and cannot be conducted at POC. Recently, electrochemical sensors [25,26,27], surface plasmon resonance (SPR) sensors [28] and impedimetric biosensors [29] have been introduced into cortisol investigation and achieved great progress, even though these technologies require mediators, complex electrode fabrication, indirect cortisol measurement, high-voltage investigation, or modification of the analyte itself. Generally, extensive efforts regarding high-sensitivity cortisol estimation via electrochemical immunosensors have been published and excellent detection limits of 1 pM to 1 μM have been obtained by the sensors [18,25,26,30,31,32]. In these studies, different dimensions of nanomaterials have been utilized in the electrochemical sensors to improve the biocompatible, electroactive and biologically functional immobilization matrices. For example, two-dimensional (2D) materials like graphene oxide (GO) sheets [33,34], layered transition metal dichalcogenides (TMDs) have been extensively studied during the past two decades [35]. In particular, molybdenum disulfide (MoS_2_), a typical member of layered transition-metal dichalcogenide, comprising a metal Mo layer sandwiched between two S layers and stacked together by weak van der Waals interactions, is drawing great attention in the electrochemical field due to its outstanding mechanical, catalytic, optical, and electric transportation properties, just as in the field of optics [36,37,38,39]. The MoS_2_ sheet with thiol groups on the surface was employed to chemically combine with noble nanomaterials for the improvement of conductivity, taking advantage of its property of large surface area, so as to be fabricated with biocomponents for biosensors. Noble metal nanoparticles possess remarkable properties, such as superior stability, high catalytic activity, simple synthesis process, bio-compatibility and easy storage, which have attracted extensive attention in biosensing electrochemical systems. In addition, combination of nanomaterials and MoS_2_ results in large electro-active surface area to volume ratio, accelerated electron transfer from electrode to the modified biomaterial, and low background current, providing a multifunctional sensing platform that combines the best features of the two materials [38,40,41,42]. Moreover, their immobilization on the disposable screen-printed electrodes offers an excellent option for cortisol detection at point-of-care testing (POCT).

In this paper, a biosensor of cortisol antibody conjugated to gold nanoparticles/molybdenum disulfide/gold nanoparticles (AuNPs/MoS_2_/AuNPs)-modified electrodes through a layer-by-layer self-assembly method was proposed for direct detection of the cortisol molecule via resistance changes of the sensor. The biomodified electrodes showed high sensitivity in a specific linear range and great specificity to cortisol, which sufficed the following portable system detection. A smartphone-controlled DPV system was developed for non-invasive salivary cortisol detection in POCT. The detection circuit was applied to convert the biochemical interaction to measurable electrical signals and the signals were transmitted to a smartphone-controlled system through Bluetooth and plotted on an app in the smartphone in real time. The smartphone-controlled system composed of the biosensor, the detection circuit and the app, and successfully quantified immunoreactions of the cortisol and distinguished cortisol from the other substances in saliva. Finally, the system was introduced to detect cortisol concentration in human saliva and the corresponding value was shown on the app. The detection results were consistent with the trend of cortisol in human saliva during a day. Thus, it was proved that the AuNPs/MoS_2_/AuNPs-fabricated biosensing system based on the smartphone-controlled DPV detector could be used for non-invasive salivary cortisol detection at POC with high sensitivity and specificity.

## 2. Methods Workflow

### 2.1. Materials and Reagents

Cortisol antibody (CORT-Ab) and cortisol was purchased from Abcam (Shanghai) Trading Co., Ltd. Sodium chloride (NaCl) and glucose were purchased from Sinopharm Chemical Reagent Co., Ltd. Uric acid (UA), glutamic acid, cysteine, bovine serum albumin (BSA) and corticosterone were from Aladdin reagent (Shanghai) Co., Ltd. Molybdenum disulfide dispersions (MoS_2_, 1 mg/mL) were obtained from Nanjing Xianfeng Nano Material Technology Co., Ltd. Ethanol, sodium sulfate solution (NaOH), polyvinyl butyral resin BUTVAR B-98 (PVB), block polymer PEO-PPO-PEO (F127), multiwall carbon nanotubes, methanol, chloroauric acid (HAuCl_4_), ferricyanide/ferrocyanide (K_4_[Fe(CN)_6_]/K_3_[Fe(CN)_6_]), phosphate-buffered saline (PBS), trisodium citrate (Na_3_C_6_H_5_O_7_), 1-ethyl-3-(3-dimethylaminopropyl) carbodiimide (EDC), N-hydroxysuccinimide (NHS), and α-thiol-ω-carboxy poly (ethylene glycol) (MW 2100 Da, COOH–PEG–SH in short) for cortisol antibody immobilization and ethanolamine (EA) were all of analytical grade and purchased from Sigma-Aldrich Co., LLC, San Luis, MO, USA. Screen-printed electrode (SPE) was purchased from GSI Technology Co., Ltd, USA.

### 2.2. Fabrication of the Sandwich-Structured Immunosensor for Cortisol Detection

**Figure 1:**
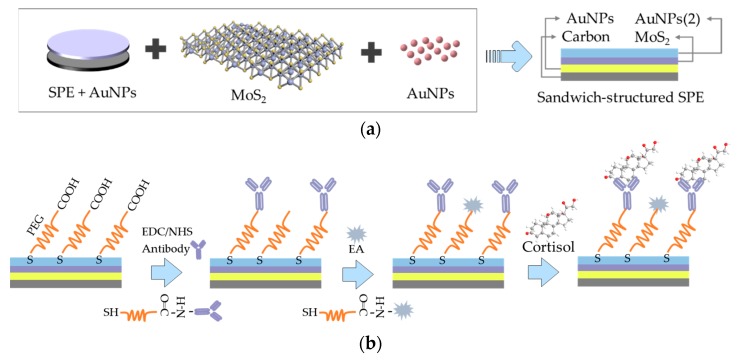
The construction and detection procedure of cortisol immunosensor. (**a**) Fabrication of layer-by-layer self-assembled gold nanoparticles/molybdenum disulfide/gold nanoparticles (AuNPs/MoS_2_/AuNPs) sandwich-structured screen-printed electrode (SPE), (**b**) covalent immobilization of cortisol antibody through self-assembled monolayer of polyethylene glycol (PEG).

Specially designed sandwich-structured immunosensors were constructed to measure current changes when cortisol antibody was binding with cortisol. Figure 1 schematically describes the fabrication of layer-by-layer self-assembled AuNPs/MoS_2_/AuNPs sandwich-structured electrodes and the covalent immobilization of cortisol antibody. The SPEs, composed of working electrode (carbon), auxiliary electrode (carbon) and reference electrode (silver/silver chloride), were applied as substrates of the immunosensors. Prior to surface modification, the electrodes were rinsed thoroughly with ethanol solution (ethanol: deionized water, 1:1) for the removal of organic residues from the electrode surface. Then, the electrodes were activated with NaOH (0.1 M) by CV over the potential range of −1 to 1 V to obtain the best state of the electrodes. The reference electrode was coated with 2 μL PVB solution, which was prepared by dissolving PVB and NaCl into methanol (79.1 mg, 50 mg, 1 mL). In order to minimize the potential drift, 2 mg F127 and 0.2 mg of multiwall carbon nanotubes were added into the PVB solution [43]. Next, the first layer of gold nanoparticles (AuNPs) were obtained by reduction of chloroauric acid (HAuCl_4_, 1%) with CV from 0 to −2 V. The working electrode turned brick red. Then, 10 μL of molybdenum disulfide dispersion (MoS_2_, 1 mg/mL) was dropped evenly onto the working electrode surface and air-dried for about 1 h to form stable Au–S covalent bonds, and an effective self-assembled monolayer (SAM) of MoS_2_ was created. After that, the layer-by-layer self-assembled sandwich-structured screen-printed electrodes (Figure 1a) were established by subsequently adding 10 μL gold nanoparticles (AuNPs) to the working electrode with linkage of Au–S bonds between MoS_2_ SAM and AuNPs, and the second self-assembled monolayer of AuNPs was formed. The preparation of the latter layer of gold nanoparticles (AuNPs) has been described in the previous papers [14,38]. In short, 100 µL chloroauric acid (HAuCl_4_, 1%) was diluted 10 times with deionized water and heated to 80 °C with constant stirring. Then 185 µL trisodium citrate (Na_3_C_6_H_5_O_7_, 1%) was quickly added for the reduction of HAuCl_4_ with the heat lasting for 60 min at 80 °C and the stir kept another 15 min for cooling. So far, AuNPs solution was obtained after centrifugation at 12,000 r.p.m for 30 min and stored in the dark for redispersion at 4 °C.

After that, the layer-by-layer self-assembled sandwich-structured working electrodes were incubated with 10 μL SH-PEG-COOH solution (1.5 mg/mL, ethanol: deionized water (DI) = 3:7) for approximately 12 h, forming stable Au-S covalent bonds between thiol group of SH-PEG-COOH and the second layer of AuNPs of the transducer and another robust self-assembled monolayer (SAM) for antibody conjugation [44,45,46]. The PEG SAM was created on the electrodes after the unbound SH-PEG-COOH was washed away with DI water. The cortisol monoclonal antibody (CORT-Ab, Abcam Co., 10 μg/mL) in PBS was conjugated onto the PEG SAM on the electrode surface with the help of EDC/NHS. 6 µL of 0.1 mM EDC in DI water was added to the working electrode and incubated for 15 min, offering a stable environment for the NHS. Successively, 0.15 mM NHS was added and incubated for 30 min to activate the carboxyl group of SH-PEG-COOH. Then, 10 µL of 10 µg/mL CORT-Ab solution was added to the electrode for 4 h incubation to form amide linkages between the amine group of CORT-Ab and carboxyl group of SH-PEG-COOH. After rinsing with DI water to eliminate the unreacted EDC/NHS solutions and unbound biomolecules, the electrode with CORT-Ab was treated with 10 µL of ethanol amine (EA) for block of non-specific protein binding, incubated for 30 min and rinsed with DI water (Figure 1b). The fabricated EA/CORT-Ab/SH-PEG-COOH/AuNPs/MoS_2_/AuNPs electrodes were stored in 4 °C for further experiments.

### 2.3. Architecture of the Smartphone-Controlled Detection Circuit and App

**Figure 2:**
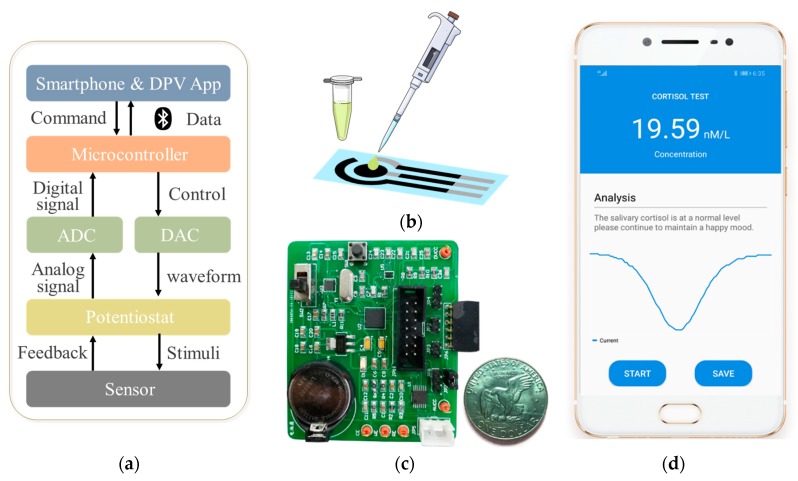
Structure of the miniaturized differential pulse voltammetry (DPV) system. (**a**) The diagram of the system, (**b**) EA/CORT-Ab/SH-PEG-COOH/AuNPs/MoS_2_/AuNPs biofunctionalized SPE/AuNPs/MoS_2_/AuNPs biofunctionalized SPE, (**c**) image of the designed printed circuit board (PCB), (**d**) the interface of the DPV detection application in the smartphone.

A miniaturized battery-powered printed circuit board (PCB) was utilized to operate the special designed immunosensor with DPV analysis. Figure 2 describes the system structure and the concrete functions from electrochemical transduction to signal transmission and graphic display on the Android smartphone screen. The smartphone-controlled DPV system was mainly composed of the modified bioelectrode sensing module (Figure 2b), an extremely compact designed printed circuit board (PCB, 6.5 cm × 5.5 cm, Figure 2c) and an App on the smartphone (Figure 2d). The specific detection circuit created the link between the bioelectrode and the smartphone control system, and it included microcontroller (MCU, MSP430), digital analog converter (DAC, DAC8562), analog digital converter (ADC, AD8608), potentiostat module and bluetooth module (HC-06 shield). The diagram is shown in Figure 2a. The MCU received control commands from the smartphone via the serial port connected with the Bluetooth module. These commands included some initial setup about pulse voltage and scanning period of stimuli. The I^2^C port of the printed circuit board would then communicate with the DAC and the digital signals would be transformed into analog form. The potentiostat module was applied to amplify the converted analog waveform for precise measurement, monitor feedback signals from the reference and counter electrodes of the printed electrodes and sent them to the ADC module. Thus, analog signals were sent out to the working electrode of modified electrodes as stimuli. The sensor transformed the chemical reaction signals into measurable electrical form. After the electrical signals were filtered by the potentiostat, the feedback signals were sent to the ADC, recorded by the printed circuit board through the I^2^C port and sent to the smartphone through Bluetooth. Eventually the feedback signals were transformed to current value through the APP and the current curve would be displayed on the smartphone screen in real-time.

The application program (app) developed on the Android smartphone was applied to control DPV detection, receive feedback signals and plot the real-time results on screen. As shown in Figure 2d, three kinds of elements were included: concentration display with analysis, graph of real-time current curve and two buttons of ‘start’ and ‘save’, aiming to start or terminate the program and save data, respectively.

### 2.4. Electrochemical Detection of Cortisol

The electrochemical workstation (CHI660e, Shanghai Chenhua Instrument Co., Ltd, Shanghai, China) was applied to perform the characterization of layer-by-layer self-assembled immunosensor via CV (−0.1–0.5 V) and electrochemical impedance spectrum (EIS), which was operated at equilibrium potential without external biasing in the frequency range of 0.1–10^5^ Hz with 10 mV amplitude. CV and EIS were all conducted in the aqueous PBS solution (10 mM, pH 7.0) containing a mixture of 5 mM K_4_[Fe(CN)_6_]/K_3_[Fe(CN)_6_] (1:1) as redox couple.

Cortisol solution of 0.5, 1, 2, 4, 10, 20, 40, 100 and 200 nM (salivary cortisol range: 2.78-22.1 nM, cortisol MW: 362.46 g/mol) were obtained by diluting cortisol in 10 mM PBS and ethanol as cosolvent. DPV was carried out with Chenhua electrochemical workstation and the smartphone-controlled DPV system in a mixed solution of 100 µL of 5 mM K_4_[Fe(CN)_6_]/K_3_[Fe(CN)_6_] (1:1) and 50 µL of cortisol solution at different concentrations, including blank PBS (10 mM) control. In DPV detection, the potential range was set from −0.1 V–0.5 V with an amplitude of 50 mV, and step voltage and scanning period were 5 mV and 0.5 seconds respectively. After addition of cortisol of different concentrations, the biosensor was washed by DI water to eliminate the unbound residual cortisol. The peak current of detection was normalized from the blank and the obtained current (ΔI) values were given to calculate the cortisol concentrations. In addition, for human salivary cortisol determination, human salivary samples were collected from volunteers. In the experiment of cortisol changes during a day, we collected 10mL saliva samples from one participant at 0 am, 6 am, 9 am, 12 pm, 3 pm, 6 pm, 9 pm. In the comparative experiment of three samples, we respectively collected 10mL samples from three participants at 9am. The supernatant was stored at −20 °C after centrifugation for 30 min at 12,000 r.p.m. to remove macromolecular protein contaminants.

Sodium chloride (NaCl), glucose (Glc), uric acid (UA), glutamate (Glu), cysteine (Cys), bovine serum albumin (BSA), corticosterone and cortisol were diluted to a final concentration of 10 nM in phosphate buffer solution. They were applied for specificity verification on a conventional electrochemical workstation and the smartphone-controlled DPV system. The detection parameters and procedure of these solutions were the same as that of cortisol. All electrochemical detections were implemented at room temperature (22 °C).

## 3. Results and Discussion

### 3.1. Characterization of the Gold Nanoparticles/Molybdenum Disulfide/Gold Nanoparticles (AuNPs/MoS_2_/AuNPs) Sandwich-Structured Immunosensor

**Figure 3:**
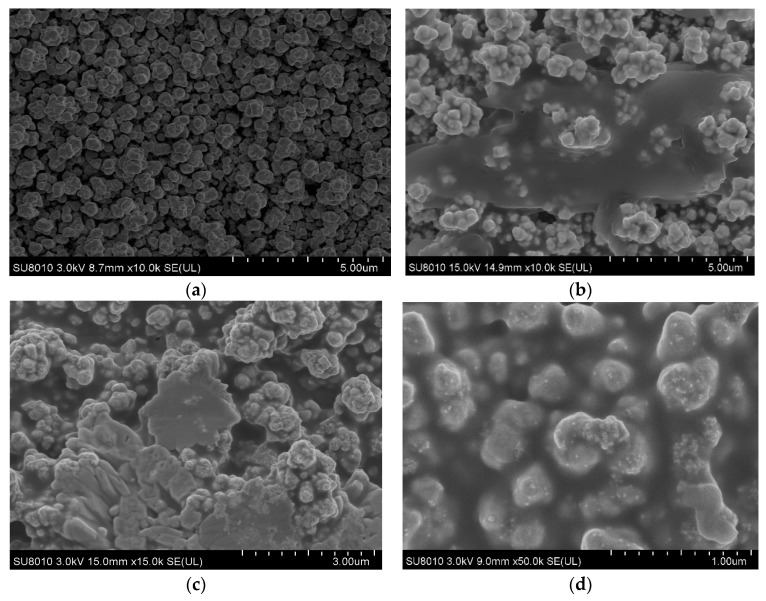
The scanning electron microscope (SEM) image of (**a**) gold nanoparticles (AuNPs with diameter of approximately 500 nm) modified SPE, (**b**) molybdenum disulfide/gold nanoparticles (AuNPs/MoS_2_)-modified SPE, (**c**) sandwich structured gold nanoparticles/molybdenum disulfide/gold nanoparticles (AuNPs/MoS_2_/AuNPs) electrode and (**d**) image of the electrode magnified 50 k times.

**Figure A1:**
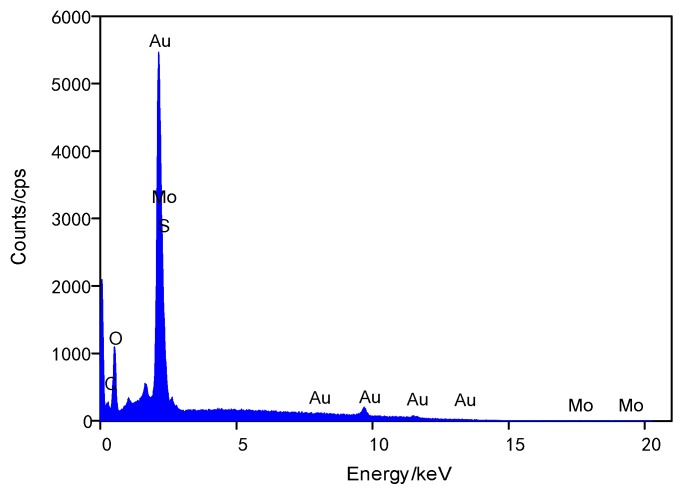
Energy-dispersive X-ray spectroscopy (EDS) image of AuNPs/MoS_2_/AuNPs-modified screen-printed electrode.

The morphology of gold nanoparticle/molybdenum disulfide/gold nanoparticle sensing platform was characterized by scanning electron microscope (SEM). It was conducted on HITACHI UHR FE-SEM SU 8010 at an accelerating potential of 3 kV and 15 kV. Figure 3a shows the SEM image of the first layer of gold nanoparticles, demonstrating the stacked, closely connected and pebble-liked gold nanoparticles. It can clearly be observed that plenty of gold nanoparticles with diameter of approximately 500 nm stay closely to each other, suggesting that the surface of screen-printed electrodes have been completely covered with gold nanoparticles. Figure 3b illustrates the molybdenum disulfide covered gold nanoparticles, where it can clearly be noticed that the gold nanoparticles have been wrapped with a membrane and the gaps between the nanoparticles were filled with monolayer molybdenum disulfide. This can provide more electrochemical active centers, helping the electrode surface to further bind more gold nanoparticles, and further expand the specific interface to bind with the antibody, so as to increase the directional adsorption of antigen and improve the detection sensitivity. Figure 3c,d displayed the structure of the prepared gold nanoparticles on the molybdenum disulfide/gold nanoparticle-modified electrode. It was observed that the second layer of gold nanoparticles was well dispersed on the molybdenum disulfide with an average size of about 30 nm. Moreover, energy-dispersive X-ray spectroscopy (EDS) was also employed to verify the structure of AuNPs/MoS_2_/AuNPs (Figure A1). Elements of gold, sulfur and molybdenum appeared in selected area. These results revealed that the layer-by-layer self-assembled AuNPs/MoS_2_/AuNPs electrode has been successfully constructed.

**Figure 4:**
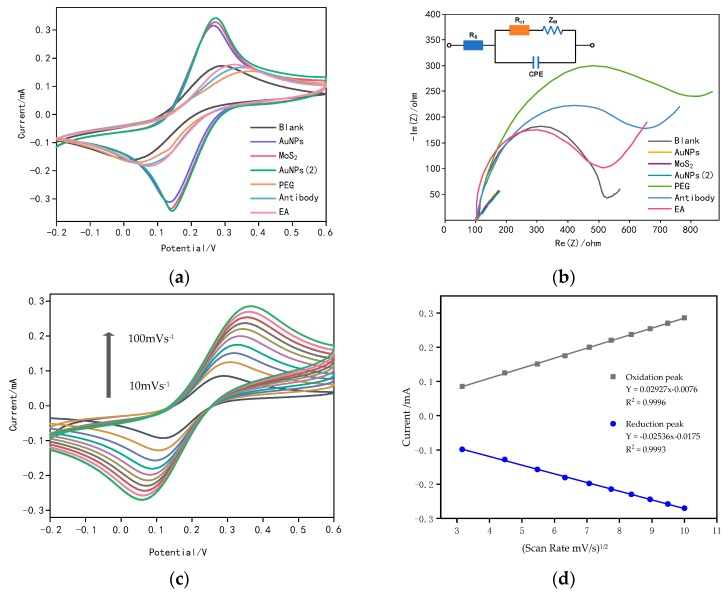
Characterization of the prepared immunosensor. (**a**) cyclic voltammetry (CV) curves of the proposed EA/CORT-Ab/SH-PEG-COOH/AuNPs/MoS_2_/AuNPs immunosnsor, (**b**) impedance spectra of AuNPs/MoS_2_/AuNPs-modified SPE, (**c**) CV curves of the proposed immunosensor in 5 mM redox couple with different scan rates (from inner to outer: 10 mVs^−1^–100 mVs^−1^), (**d**) the dependence of redox peak currents vs. the square-root of the scan rate..

To further describe the electrochemical performance of the stepwise modified electrochemical immunosensor, CV and electrochemical impedance spectroscopy (EIS) were employed at each step during modification of the electrodes and functionalization with cortisol antibody for biosensing. Figure 4a shows CV curves of electrodes with different fabrication. The changes of CV peak currents that represent different modifications under the same conditions reveal the conversion of the electrode surface. The peak current of the CV curves increased from 171 μA to 316 μA, corresponding to the bare carbon screen-printed electrode modified with first layer of AuNPs which was reduced from HAuCl_4_. With the dispersion of molybdenum disulfide (MoS_2_) on the AuNPs-modified electrodes, solid gold–sulfur bonds were formed and the current response continued to rise. The peak current increased to 346 μA when the as-prepared gold nanoparticles were added to the electrode surface. Except for changes in current, potential difference between oxidation peak and reduction peak decreased by 147 mV compared with the bare electrode. These results revealed that the electrical conductivity and electrochemical reversibility were clearly improved. This demonstrated that the layer-by-layer self-assembled AuNPs/MoS_2_/AuNPs sandwich-structured electrodes could optimize the electrocatalytic properties and electrochemical characteristic of the bare screen-printed electrodes. This credit could be given to the fabrication material of MoS_2_ and different size of AuNPs, large specific surface area, wonderful adhesion, robust loading and high catalytic efficiency of AuNPs/MoS_2_/AuNPs structured nanomaterial. The assembly of PEG on the electrode hindered the electron transfer process at the electrode/electrolyte interface and caused a sharp drop in current response, which affirmed the formation of PEG self-assembled monolayer (SAM). The conjugation of cortisol antibody to PEG SAM, the top layer of the modified electrode, led to an increase of current response. After ethanolamine was added to the electrode, a slight increase in peak current was observed. The ascent in current could be ascribed to the removal of unbound cortisol antibody molecule and the blockage of non-specific binding. Figure 4b illustrates the Nyquist plots of impedance spectra (EIS) investigated in each procedure during the fabrication. The Randles circuit model displayed in Figure 4b could be introduced to analyze the impedance spectrum, including solution resistance (R_s_), charge transfer resistance (R_ct_), warburg impedance (Z_w_), and constant phase element (CPE). The diameter of the semicircle portion at high frequencies that represents the electron transfer process is essential in the EIS and its value is the same as the charge transfer resistance (R_ct_) in the Randles model. The bare carbon screen-printed electrode exhibited an initial electron transfer resistance (R_ct_ = 393 Ω). After chloroauric acid (HAuCl_4_) was reduced to gold nanoparticles on the electrode, an extreme decrease in electron transfer resistance was observed with a negligible value. Successively, the electron transfer resistances of MoS_2_ and prepared AuNPs were still at a very low level. Fortunately, this result was consistent with the previous CV curves and the impedance spectra was just unable to display such a low level of electron transfer resistance, implying that the modified electrodes had excellent conductivity. However, the dramatic rise of R_ct_ to 736 Ω indicated the PEG SAM formation, which retarded the electron-transfer process. Covalent binding of the cortisol antibody to the PEG SAM resulted in the decrease of R_ct_ to 536 Ω. The R_ct_ continued to drop after incubation of EA. The EIS results are in accordance with those obtained by CV, demonstrating that the electrodes have been successfully biofunctionalized with cortisol antibody for cortisol detections.

Figure 4c exhibits the CV curves, describing electrochemical behavior of the constructed immunosensor, mainly the influence caused by scan rates under the same conditions. The peak currents of oxidation and reduction increase as the scan rates increase from 10 to 100 mV/s^−1^ and the well-defined redox peaks as a function of scan rates (R_o_^2^ = 0.9762, R_r_^2^ = 0.9717) can be observed in Figure 4d, indicating that it is a controlled by a diffusion electrocatalytic process and the immunosensor is very electrochemically active [27,47,48].

### 3.2. Cortisol Detection Based on the Smartphone System

**Figure 5:**
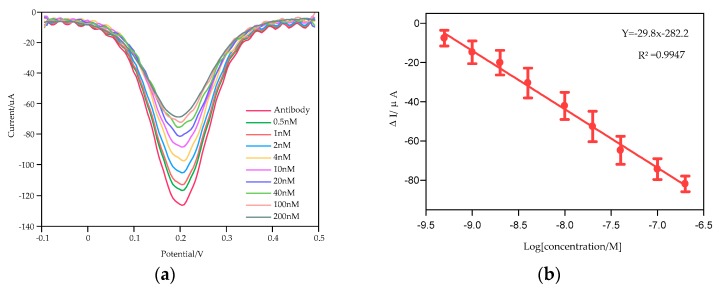
Electrochemical detection of cortisol using smartphone-controlled DPV system. (**a**) DPV responses of cortisol from 0.5 nM to 200 nM, (**b**) calibration curve for cortisol immunosensor.

**Figure A2:**
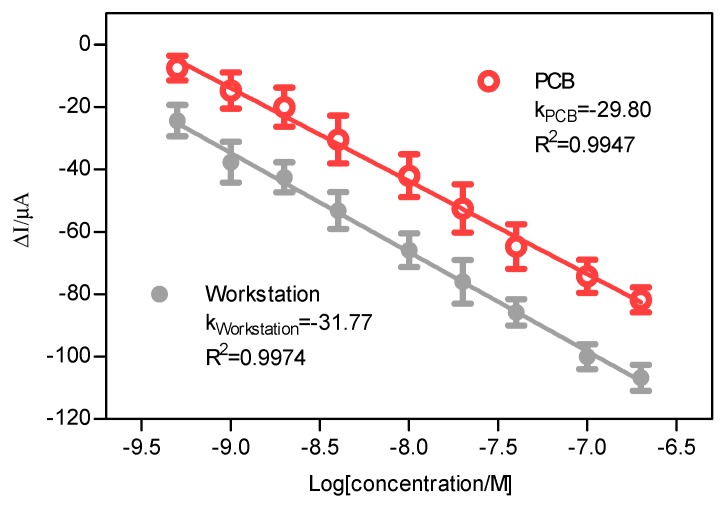
Calibration curves for cortisol immunosensor using smartphone-controlled DPV system (red line) and the electrochemical workstation (grey line).

The miniaturized smartphone-controlled DPV system was applied to detect cortisol at POC with the biofunctionalized screen-printed electrodes which possess good electrocatalytic properties. As shown in Figure 5a, the oxidation current response was found to increase to a maximum at around 0.2 V upon voltage sweeping from −0.1 V to 0.5 V. When cortisol was conjugated on the surface of the modified immunosensor, the peak current levels were proved to decrease in a sequence as the cortisol concentration increases from 0.5 nM to 200 nM. This result was probably attributed to the insulating immune complex formed between cortisol and the antibody and more cortisol molecules captured in the immunological binding process. It hinders electron transfer, causing an increase in impedance of the sensitive layer on the surface of the electrode. Thus, the electron transfer rate was slowed down and the peak current dropped. Figure 5b presents the calibration curve of the cortisol ranging from 0.5 to 200 nM, generated during the process of binding with the cortisol antibody and normalized with the cortisol-free biofunctionalized electrode. The linear relationship between the normalized current and the logarithm of cortisol concentration was fitted into the equation as y = −29.8 log(x) – 282.2 with a correlation coefficient of 0.9947, where x and y represent cortisol concentration (M) and current change ΔI (μA), respectively. The sensitivity was estimated to be about 30 μA M^−1^. The detection limit of the prepared immunosensor was estimated as 0.11 nM using 3σ/s calculation, where σ is the standard deviation of the baseline signal and s is the slope of the calibration graph. It is demonstrated that biological combination between cortisol and cortisol antibody could be sensitively investigated by the miniaturized smartphone-based DPV system with the layer-by-layer self-assembled EA/CORT-Ab/SH-PEG-COOH/AuNPs/MoS_2_/AuNPs immunosensor, which sufficed the sensitivity requirement of the smartphone-controlled DPV system. The contrast of current response changes induced by the workstation and the miniaturized smartphone-based DPV system was presented in Figure A2. It can be observed that the workstation exhibited better performance than the miniaturized DPV system, but the latter possesses advantages from the perspective of portability, real-time detection, low cost and a detection performance of linearity and specificity. The linear range detected was from 0.5 nM to 200 nM, sufficient for the detection of real salivary concentration range in healthy human body (1–8 ng/mL, that is 2.78–22.2 nM), indicating that the EA/CORT-Ab/SH-PEG-COOH/AuNPs/MoS_2_/AuNPs modified immunosensor could provide satisfying electrochemical performance for the miniaturized smartphone-controlled DPV system, and the DPV platform integrating with the special modified immunosensor could offer a promising approach for non-invasive real human salivary cortisol detection with excellent performance at POC. 

### 3.3. Specificity and Stability Study

**Figure 6:**
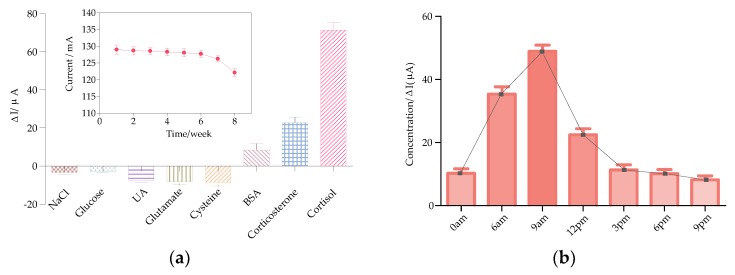
(**a**) Selective responses of cortisol and substances existed in saliva using the smartphone-controlled DPV system, inset shows the stability results of the immunosensor, (**b**) DPV responses of the variation of cortisol level in human saliva during a day based on the smartphone system.

**Figure A3:**
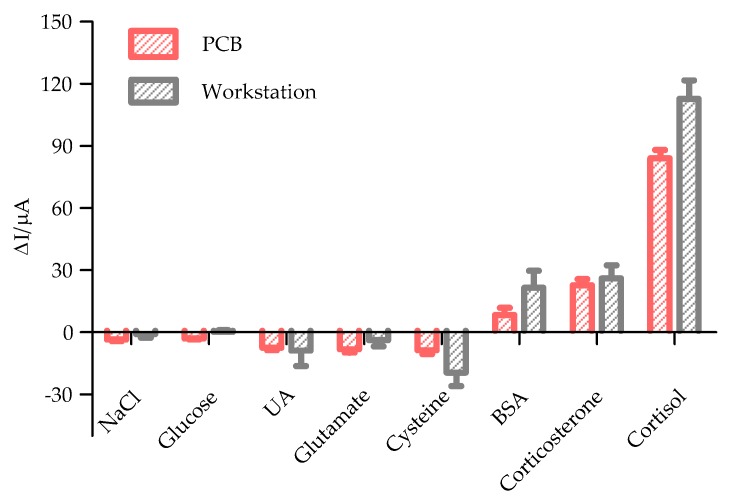
Selective responses of cortisol and substances existed in saliva using the smartphone-controlled DPV system (red box) and the electrochemical workstation (grey box).

To evaluate the specificity characteristic of the biofunctionalized immunosensors for cortisol, the developed immunosensor was investigated with several common substances in saliva, such as sodium chloride, glucose, uric acid, glutamic acid, cysteine, bovine serum albumin and corticosterone. Sodium chloride and glucose are two basic ions in saliva. Uric acid is an electrochemically active substance existing in saliva which may influence the detection of cortisol. Glutamic acid and cysteine are important amino acids in the human body. Bovine serum albumin is detected here as a representative of macromolecular biological proteins and corticosterone is a derivative of cortisol that has a similar molecular structure with cortisol. As shown in Figure 6a, cortisol is the only substance that caused a dramatic normalized current response while the other investigated interferents consistently exhibited relatively low response current changes, except for that the corticosterone induced a small response which was still far from the signal caused by cortisol. In addition, the current changes detected by the miniaturized smartphone-controlled DPV system were consistent with that of the workstation (Figure A3), which proved once again that the miniaturized smartphone-controlled DPV system is competent in detecting cortisol at POC, eliminating the need to use the bulky traditional workstation. Most importantly, it is verified that the EA/CORT-Ab/SH-PEG-COOH/AuNPs/MoS_2_/AuNPs fabricated immunosensor is highly specific and selective to cortisol, thus it can meet the requirement of portable system detection and distinguish cortisol from the other interferents. The inset picture in Figure 6a exhibits the stability of the prepared immunosensor tested by the DPV system. It can be seen that the current response remained stable until being stored at 4 °C for six weeks. After that, the magnitude of the current response dropped by 6% in the seventh week and 10% in the eighth week. These results demonstrated that the proposed immunosensor have great potential for noninvasive real human salivary cortisol detection at POC with the smartphone-controlled differential pulse voltammetry system.

### 3.4. Human Saliva Test Using the Smartphone-Controlled Differential Pulse Voltammetry (DPV) System

For the sake of verifying the practicality of the proposed cortisol detection strategy, the fabricated immunosensor was investigated for human salivary cortisol test with the smartphone-controlled DPV system. Table 1 describes the estimation results of human salivary cortisol. The saliva samples were collected from three healthy adults at 9 a.m. on the same day and then spiked with different concentrations (10 nM, 20 nM, 40 nM) of cortisol standard solution, following the principle of the internal standard method. The value of cortisol concentration and the detection plot were presented on the screen of the smartphone with Android application (Figure 2d). A recovery ratio could be adopted to evaluate the performance of analytical methods and the accuracy of the measurement system. The recovery of cortisol from three subjects is in the range of 96.30%–105.64%. This indicated that the proposal was applicable for cortisol detection in real saliva. To further examine the applicability and practicability of the whole system, the variation of cortisol level in human saliva during a day (n = 3) was monitored (Figure 6b). It is clearly revealed that cortisol reached the highest level for a period of time after rising and then gradually decreased to a relatively low level, which is in consistent with previous research [11,12]. It is suggested that the developed EA/CORT-Ab/SH-PEG-COOH/AuNPs/MoS_2_/AuNPs electrochemical immunosensor integrating with the miniaturized DPV system possesses striking features for non-invasive quantification of cortisol in human saliva at POC.

## 4. Conclusions

In this paper, an ultrasensitive immunosensor has been successfully fabricated for non-invasive salivary cortisol detection at POC using a miniaturized smartphone-controlled DPV system. In order to meet the detection requirements of a miniaturized DPV system, the layer-by-layer self- assembled sandwich-structured transducer of AuNPs/MoS_2_/AuNPs based on a screen-printed electrode was constructed via a robust gold–sulfur bond, offering large surface area and excellent biocompatibility. Then, cortisol antibody was immobilized on the sandwich-structured electrode through the self-assembled monolayer of PEG. Non-specific binding was hampered by ethanolamine. With the miniaturized DPV system integrating with the immunosensor, a portable detection system was obtained and it was utilized for both standard cortisol and human salivary cortisol detection. The results verified the excellent performance of the immunosensor system from aspect of sensitivity during a specific range from 0.5–200 nM. Moreover, the immunosensor possesses favorable specificity, stability and portability, demonstrating the great potential application of the immunosensor system for non-invasive human salivary cortisol detection and modern stress and fatigue monitoring at POC.

## Figures and Tables

**Table 1:** Determined results of salivary cortisol from three subjects and recoveries of samples spiked with different concentration of cortisol using the smartphone-controlled DPV system.

Samples	Added (nM)	Found (nM)	Recovery (%)
Sample 1	0	19.59	-
	10	29.72	101.29
	20	39.21	98.08
	40	61.39	104.51
Sample 2	0	8.20	-
	10	18.03	98.23
	20	28.17	99.84
	40	50.46	105.64
Sample 3	0	14.33	-
	10	24.57	102.43
	20	33.59	96.30
	40	54.29	99.88
